# Prevalence of Dyslipidemias in Three Regions in Venezuela: The VEMSOLS Study Results

**DOI:** 10.5935/abc.20170180

**Published:** 2018-01

**Authors:** Juan P. González-Rivas, Ramfis Nieto-Martínez, Imperia Brajkovich, Eunice Ugel, Alejandro Rísquez

**Affiliations:** 1 The Andes Clinic of Cardio-Metabolic Studies, Miami, FL - USA; 2 Geriatric Research, Education and Clinical Center (GRECC) and South Florida Veterans Affairs Foundation for Research & Education, Miami VA Healthcare System, Miami, FL - USA; 3 Department of Physiology - School of Medicine - University Centro-Occidental “Lisandro Alvarado” and Cardio-metabolic Unit 7, Barquisimeto, Venezuela; 4 Department of Internal Medicine B - School of Medicine “Luis Razetti” - University Hospital of Caracas - Universidad Central de Venezuela; 5 Department of Preventive Medicine - School of Medicine - Universidad CentroOccidental “Lisandro Alvarado”; 6 Department of Social and Preventive Medicine - School of Medicine, Universidad Central de Venezuela

**Keywords:** Dyslipidemias / epidemiology, Cardiovascular Diseases, Risk Factors, Stroke / mortality, Obesity, Metabolic Syndrome

## Abstract

**Background:**

The prevalence of dyslipidemia in multiple regions of Venezuela is unknown. The Venezuelan Metabolic Syndrome, Obesity and Lifestyle Study (VEMSOLS) was undertaken to evaluate cardiometabolic risk factors in Venezuela.

**Objective:**

To determine the prevalence of dyslipidemia in five populations from three regions of Venezuela.

**Methods:**

During the years 2006 to 2010, 1320 subjects aged 20 years or older were selected by multistage stratified random sampling from all households in five municipalities from 3 regions of Venezuela: Lara State (Western region), Merida State (Andean region), and Capital District (Capital region). Anthropometric measurements and biochemical analysis were obtained from each participant. Dyslipidemia was defined according to the NCEP/ATPIII definitions.

**Results:**

Mean age was 44.8 ± 0.39 years and 68.5% were females. The prevalence of lipids abnormalities related to the metabolic syndrome (low HDL-c [58.6%; 95% CI 54.9 - 62.1] and elevated triglycerides [39.7%; 36.1 - 43.2]) were the most prevalent lipid alterations, followed by atherogenic dyslipidemia (25.9%; 22.7 - 29.1), elevated LDL-c (23.3%; 20.2 - 26.4), hypercholesterolemia (22.2%; 19.2 - 25.2), and mix dyslipidemia (8.9%; 6.8 - 11.0). Dyslipidemia was more prevalent with increasing body mass index.

**Conclusion:**

Dyslipidemias are prevalent cardiometabolic risk factors in Venezuela. Among these, a higher prevalence of low HDL is a condition also consistently reported in Latin America.

## Introduction

In Venezuela, cardiovascular disease (CVD), represented by ischemic heart disease (16.3%) and stroke (7.7%), was the major cause of death in 2012.^1^ Both are strongly related with modifiable risk factors. According to the INTERHEART^2^ and the INTERSTROKE^3^ studies, dyslipidemias, assessed as increased levels of apolipoprotein (ApoB/ApoA1 ratio), represented the 49.2% and the 25.9% of the attributable risk for acute myocardial infarction and stroke, respectively. Randomized controlled clinical trials have consistently demonstrated that a reduction in low-density lipoprotein cholesterol (LDL-C) with statin therapy reduces the incidence of heart attack and ischemic stroke. For every 38.6 mg/dL LDL-c reduction, the annual rate of major vascular events decreases to one-fifth.^4^

Studies evaluating the prevalence of dyslipidemias in Venezuela have been compiled.^5^ However, most of them have small samples, and only two are representative of a city or a state. In 1,848 adults from the city of Barquisimeto, in the western region of the country, the Cardiovascular Risk Factor Multiple Evaluation in Latin America (CARMELA) study^6^ reported the lowest prevalence of hypercholesterolemia (cholesterol ≥ 240 mg/dL) observed in Latin America (5.7%).^6^ In 3,108 adults from the state of Zulia, Florez et al.^7^ documented the prevalence of atherogenic dyslipidemia (high triglycerides and low levels of high- density lipoprotein of cholesterol [HDL-c]) in 24.1%. This number was higher in men than women, and increased with age. No study in Venezuela has included more than one region, prompting the design of the Venezuelan Metabolic Syndrome, Obesity and Lifestyle Study (VEMSOLS). This paper presents the results of VEMSOLS, specifically the prevalence of dyslipidemia in five populations of three regions in Venezuela.

## Methods

### Design and Subjects

An observational, cross-sectional study was designed to determine the prevalence of cardiometabolic risk factors in a sub-national sample of Venezuela. Five municipalities from three regions were evaluated: Palavecino, in Lara State (urban), from the Western region; Ejido (Merida city), in Merida State (urban), and Rangel (Páramo area), in Merida State (rural), both from the Andes region; Catia La Mar, in Vargas state (urban), and Sucre, in the Capital District (urban), both from the Capital region. From 2006 to 2010, a total of 1,320 subjects aged 20 years or more, who had lived in their houses for at least six months, were selected by a two-stage random sampling. Three different geographic regions of the country - Andes, mountains at the south; Western, llanos in the middle; and the Capital District, coast at the north - were assessed. Each region was stratified by municipalities and one was randomly selected. A map and a census of each location were required to delimit the streets or blocks, and to select the households to visit in each municipality. After selecting the sector to be surveyed in each location, the visits to households started from number 1 onwards, skipping every two houses. Pregnant women and participants unable to stand up and/or communicate verbally were excluded. All participants signed the informed consent form for participation.

The sample size was calculated to detect the prevalence of hypercholesterolemia (the lowest prevalent condition reported in Venezuela) in 5.7%^6^, with standard deviation of 1.55%, which allows to calculate the 95% confidence interval (95% CI). The minimal estimated number of subjects to be evaluated was 830. Overall, 1,320 subjects were evaluated (89.4% from the urban and 10.6% from the rural area).

### Clinical and biochemical data

All subjects were evaluated in their households or in a nearby health center by a trained health team according to a standardized protocol. Each home was visited twice. In the first visit, the participants received information about the study and signed the written informed consent form. Demographic and clinical information was obtained using a standardized questionnaire. Weight was measured with as few clothes as possible, without shoes, using a calibrated scale. Height was measured using a metric tape on the wall. Waist circumference was measured with a metric tape at the iliac crest at the end of the expiration. Body mass index was calculated (BMI: weight[kg]/height[m]^2^).

In the second visit, blood samples were drawn after 12 hours of overnight fasting. Then, they were centrifuged for 15 minutes at 3000 rpm, within 30-40 minutes after collection, and transported with dry ice to the central laboratory, where they were properly stored at -40ºC until analysis. Data from participants who were absent during the first visit were collected. Total cholesterol,^8^ triglycerides,^9^ LDL-c, and HDL-c^10^ were determined by standard enzymatic colorimetric methods.

### Categorization of variables

Dyslipidemia was defined according the National Cholesterol Education Program /Adult Treatment Panel III (NCEP/ATPIII)^11^, being categorized in 6 types. Of these, four were isolated dyslipidemias: Low HDL-c (hyperalphalipoproteinemia) < 40 mg/dL in men and < 50 mg/dL in women; high triglycerides: ≥ 150 mg/dL; hypercholesterolemia (≥ 240 mg/dL of total cholesterol); high LDL-c ≥ 160 mg/dL; and two were combined dyslipidemias: atherogenic dyslipidemia (triglycerides ≥ 150 mg/dL + low HDL-c) and mixed dyslipidemia (triglycerides ≥ 150 mg/dL + total cholesterol ≥ 240 mg/dL). Additionally, individuals were classified according to BMI as normal weight (BMI < 25 kg/m^2^), overweight (BMI ≥ 25 kg/m^2^ and < 30 kg/m^2^), or obese (BMI ≥ 30 kg/m^2^).^12^ Abdominal obesity was established by waist circumference ≥ 94 cm in men and ≥ 90 cm in women.^13^

### Statistical analysis

All calculations were performed using the SPSS 20 software (IBM corp. Released 2011. Armonk, NY: USA). It was verified that all variables had normal distribution using a normality test (Kolmogorov-Smirnov). All variables were continuous and data were presented as mean ± standard deviation (SD). Differences between mean values were assessed with the t-test. Proportions of subjects with dyslipidemia were presented as prevalence rates and 95% confidence intervals (CI). A Chi-square test was applied to compare different frequencies by gender, nutritional status and abdominal obesity. P-value of < 0.05 was considered statistically significant.

## Results

### Characteristics of the subjects

Two thirds of the study subjects were female. Men had higher triglycerides, waist circumference and lower HDL-c than women (Table 1). Age, BMI, total cholesterol and LDL-c were similar.

**Table 1:** Subject Characteristics

	Men	Women	Total	Significance
Participants (n, %)	412 (31.2)	908 (68.8)	1320 (100)	
Age (years)	45.8 ± 14.8	44.4 ± 14.0	44.8 ± 14.3	NS
Body mass index (kg/m^2^)	27.7 ± 5.0	27.6 ± 5.3	27.6 ± 5.2	NS
Waist circumference (cm)	96.6 ± 13.2	89.8 ± 12.3	91.9 ± 13.0	< 0.0001
High density lipoprotein (HDL-c) (mg/dL) *	43.2 ± 10.4	47.2 ± 10.9	45.9 ± 10.9	NS
Triglycerides (mg/dL)	175.3 ± 154.7	140.0 ± 87.3	151.0 ± 114.3	< 0.0001
Total cholesterol (mg/dL)	207.7 ± 46.5	206.3 ± 47.6	206.7 ± 47.2	NS
Low density lipoprotein (LDL-c) (mg/dL)	131.0 ± 43.4	131.4 ± 43.8	131.3 ± 43.7	NS

Data are mean ± SD. Gender differences according t-test.

### Prevalence of dyslipidemia

**Figure 1:**
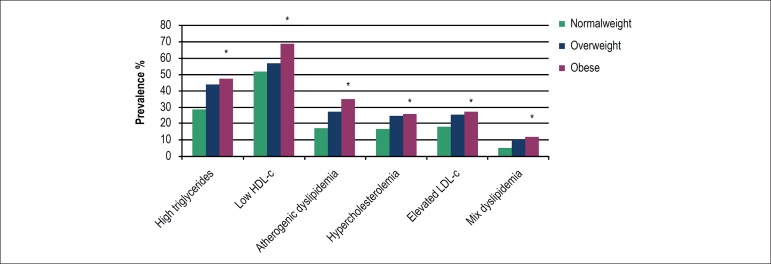
Prevalence of dyslipidemia by nutritional status. *Difference in the prevalence of dyslipidemia according to nutritional status using Chi-square (p < 0.01). High triglycerides: 150 mg/dL; low HDL-c: < 40 mg/dL in men and < 50 mg/dL in women; atherogenic dyslipidemia: triglycerides = 150 mg/dL + low HDL-c; hypercholesterolemia: total cholesterol = 240 mg/dL; elevated LDL-c: = 160 mg/dL; mixed dyslipidemia: triglycerides = 150 + total cholesterol = 240 mg/dL.

**Figure 2:**
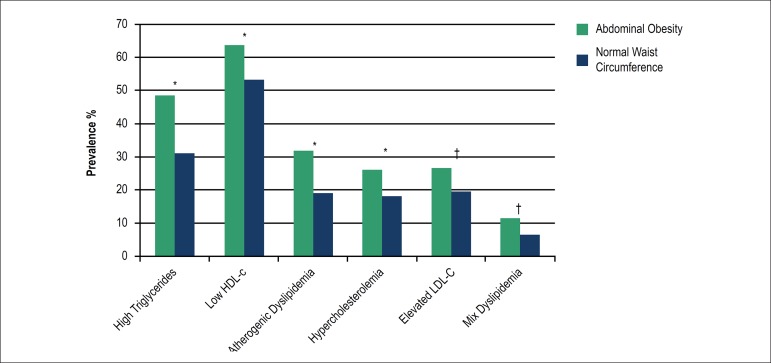
Prevalence of dyslipidemias by abdominal obesity (waist circumference = 94 cm in men and = 90 cm in women). Significant difference of the prevalence of dyslipidemia between abdominal obesity or normal waist circumference *(p < 0.001) †(p = 0.002). High triglycerides = 150 mg/dL; Low HDL-c < 40 mg/dL in men and < 50 mg/dL in women; Atherogenic dyslipidemia triglycerides =150 mg/dL + low HDL-c; Hypercholesterolemia =240 mg/dL; Elevated LDL-c = 160 mg/dL; Mix dyslipidemia triglycerides = 150 + cholesterol = 240 mg/dL.

Low HDL-c was the most prevalent lipid change present in nearly seven of ten women, and in about four of ten men (p < 0.01), followed by high triglycerides that were present in half of the men and in one third of women (p < 0.01). Their combination, atherogenic dyslipidemia, was observed in 25.9% of subjects, followed in frequency by increasing LDL-c and total cholesterol levels (Table 2). Mixed dyslipidemia was observed in only 8.9% of the subjects, and was higher among men than in women. An increasing prevalence of all types of dyslipidemias was found when individuals were classified according to BMI and at the presence of abdominal obesity (Figure 1 and Figure 2). The prevalence of hypercholesterolemia, high LDL-c and mixed dyslipidemia were similar in overweight and obese subjects, but higher than those found in the normal weight group.

**Table 2:** Prevalence of Dyslipidemias by Gender

	Men	Women	Total	Significance
	412	908	1320	
Low HDL-c (< 40 mg/dL in men and < 50 mg/dL in women)	42.2 (38.6 – 45.8)	66.0 (62.5 – 69.4)	58.6 (54.9 – 62.1)	< 0.0001
Elevated triglycerides (≥ 150 mg/dL)	49.5 (45.8 – 53.1)	35.2 (31.7 – 38.7)	39.7 (36.1 – 43.2)	< 0.0001
Hypercholesterolemia (≥ 240 mg/dL)	23.8 (20.7 – 26.8)	21.5 (18.5 – 24.5)	22.2 (19.2 – 25.2)	NS
Elevated LDL-c (≥ 160 mg/dL)	22.8 (19.8 – 25.9)	23.5 (20.5 – 26.6)	23.3 (20.2 – 26.4)	NS
Atherogenic dyslipidemia (triglycerides ≥ 150 mg/dL + low HDL-c)	25.2 (22.1 28.0)	26.2 (23.0 – 29.4)	25.9 (22.7 – 29.1)	NS
Mixed dyslipidemia (triglycerides ≥ 150 + cholesterol ≥ 240 mg/dL)	12.4 (9.9 – 14.7)	7.4 (5.5 – 9.3)	8.9 (6.8 – 11.0)	0.002

Data are showed in percentage (95% CI). Gender differences according to the Chi-square test.

## Discussion

The present study reports that the most prevalent lipid abnormality in our sub-national sample of adults in Venezuela is the low HDL-c (58.6%), followed by high triglycerides (38.7%), whereas the prevalence of hypercholesterolemia (22%) and its combination with hypertriglyceridemia (8.9%) were lower. Similar findings have been reported in earlier studies, both in Venezuela (Zulia state, Low HDL-c 65.3%, high triglycerides 32.3%),^7^ and Mexico (Low-HDL 48.4% and high triglycerides 42.3%).^14^ Using a cut-off point similar to that in our study, an extremely high prevalence of hypoalphalipoproteinemia has been also observed in Valencia city (90%)^15^ and the Junquito municipality (81.1%),^16^ both in the central region of Venezuela. Similarly to the observed in men in our study (49.5%), the aforementioned studies in Valencia and Junquito also reported high prevalence of elevated triglycerides (51%).^15,16^ Most of these results are consistent with previous findings in the Latin America region. In a systematic review of metabolic syndrome in Latin America, the most frequent change was low HDL-c in 62.9% of the subjects.^17^

Although hypercholesterolemia (22.2%) is significantly less common compared with the aforementioned alterations, it was higher than the CARMELA study (5.7%) in Barquisimeto,^6^ and similar to that observed in Valencia (19.0%).^15^ Therefore, hypercholesterolemia remains as a cardiovascular risk factor to be considered when implementing public health measures in the Venezuelan population. Other of our findings are consistent with previous studies reporting that the prevalence of dyslipidemia increases with adiposity, and subjects with overweight/obesity^14,18^ and abdominal obesity^18^ show worse lipid profiles than subjects of normal weight. As in our study, higher figures of elevated triglycerides in male,^14,18^ and no differences between overweight and obese subjects when grouped according to BMI,^14^ have been reported.

Dyslipidemias can be caused by both genetic and environmental factors (obesity, smoking, low physical activity). In our study, the prevalence of low HDL-c without other lipid abnormalities was 29.2% (male 15%, female 35.7%). Of these, those with low HDL-c and normal weight (total 10.6%, male 5.3%, female 13.0%) could suggest the proportion of cases of hypoalphalipoproteinemia that could be associated with genetic factors. Also, part of the prevalence of low HDL-c in this population can be explained by metabolic factors (i.e., insulin resistance), a condition that produces modifications in more than one lipid sub-fraction. In fact, the prevalence of atherogenic dyslipidemia (25.9%) in our study was significant and remarkably similar to that reported by Florez et al.^7^ in the Zulia region (24.1%). Atherogenic dyslipidemia is the pattern most frequently observed in subjects with metabolic syndrome and insulin resistance, and both abnormalities are components of the metabolic syndrome definition. Besides genetic or metabolic factors, environmental adverse conditions are also important in Venezuela. The factors involving nutritional transition promoted inappropriate eating and lifestyle patterns in Venezuela and other Latin American countries, clearly contributing with the incidence of non-communicable diseases, especially those related to obesity and diabetes.^19^ A follow-up survey of food consumption, based on the food purchase, reported that caloric intake and the selection of foods with lower quality have increased in Venezuela.^20^ A high rate of physical inactivity (68%) has also been reported in Venezuela in two studies involving 3,422 adults.^5^

Successful dietary strategies to reduce dyslipidemias and other metabolic syndrome components should include energy restriction and weight loss, manipulation of dietary macronutrients, and adherence to dietary and lifestyle patterns, such as the Mediterranean diet and diet/exercise.^21^ After the evaluation of the typical food-based eating and physical activity pattern in the Venezuelan population, culturally-sensitive adaptations of the Mediterranean diet with local foods and physical activity recommendations have been proposed.^5,22^ Specific recommendations for patients with dyslipidemia have been also included in local clinical practice guidelines.^23^

Some limitations can be observed in the present study. The sample did not represent the entire population of the country; only three of the eight regions of Venezuela were included. Additionally, in the VEMSOLS, eating pattern and physical activity were not investigated. The cut-off point for low HDL and triglycerides used was established for the metabolic syndrome definition, which can limit the comparison with other studies using a level below 35^14^ or 40^18^ mg/dL to define hypoalphalipoproteinemia. However, despite these limitations, this study is the first report of dyslipidemias in more than one region of Venezuela. A national survey in Venezuela in ongoing (Estudio Venezolano de Salud Cardiometabólica, EVESCAM study). Data collection will be completed in 2017.

## Conclusions

This is the first report presenting the prevalence of dyslipidemia in more than one region of Venezuela. The results observed are consistent with other Latin American studies, reporting low HDL-c as the most frequent lipid alteration in the region. Additionally, high levels hypercholesterolemia were observed. Both conditions could be related with CVD, which represent a major public health problem in the region. A suggestion resulting from our findings is to monitor a complete lipid profile during medical check-ups, because in some Latin-American countries it is common to check only total cholesterol. The triggers of these changes need to be determined in future studies. The implementation of strategies focused in proper nutrition, more physical activity and avoiding weight gain is imperative.
